# Society Proceedings

**Published:** 1912-09

**Authors:** 


### SOCIETY PROCEEDINGS.

---

The 14th Annual Meeting of the *American Hospital Association* will be held in the Hotel Ponchartrain, Detroit, Tues. to Friday, Sept. 24-27, 1912. Various phases of business management, philanthropy, nursing, etc., will be discussed. Dr. R. R. Ross of the Buffalo General Hospital will read on the Use of Salvarsan. The Secretary is J. N. E. Brown, M. B., 90 Charles St., East, Toronto, Ont.

---

Buffalo Academy of Medicine Officers 1912-13: President, Dr. W. Scott Renner; Treasurer Dr. Lawrence Hendee; Secretary, Dr. Harry R. Trick; Trustee for one year, Dr. Thos. F. Dwyer; Trustee for two years, Dr. John H. Pryor; Trustee for three years, Dr. James W. Putnam. Medical Section. Chairman, Dr. Carl S. Tompkins; Secretary, Dr. Herman K. DeGroat. Surgical Section. Chairman, Dr. Herbert A. Smith; Secretary, Dr. Chas. W. Bethune. Obstetrical and Gynecological Section. Chairman, Dr. Frank W. Ransom; Secretary, Dr. Descum E. McKenny. Pathological Section. Chairman, Dr. Guy L. McCutcheon; Secretary Dr. A. A. Thibaudeau. Dr. Stephen Y. Howell, retiring president, ex-officio member of the Council.

In 1911-12, the Council held ten meetings with an average attendance of nine.

The Academy held four Stated Meetings as follows:

| | |
|---|---|
| Oct. 3, 1911. Program by Surgical section, attendance .... | 80 |
| Dec. 19, 1911. Program by Obst. and Gyn. sec., attendance .. | 65 |
| Feb. 27, 1912. Program by Pathological sec., attendance .... | 75 |
| Mch. 12, 1912. Program by Medical section, attendance .... | 75 |
| Nov. 4, 1911. W. J. Mayo, attendance .................... | 254 |
| May 21, 1912. Special Meeting, attendance .............. | 50 |
| Number of Fellows in good standing June 1, 1911 ...... | 359 |

New members received during the year, Resident, 10, Non-resident, 3, total, 13; Members lost during the year, by death, 2, by resignation, 0, Dropped for non-payment of dues, 30, Total, 32, net loss 19.

Number of members in good standing June 1, 1912 ...... 340

Respectfully submitted, HARRY R. TRICK, *Secretary.*

The first meeting of the season 1912-13 will be held at the Public Library Building, Tues. Oct. 1, at 8.30 P. M., under the direction of the Surgical Section. Program to be announced later.

---

The Elmira Academy of Medicine will hold its first meeting of the season on Sept. 4. Dr. R. P. Bush will read a paper on "The County Tuberculosis Sanatoria."

